# Advances in Phytonanotechnology: A Plant-Mediated Green Synthesis of Metal Nanoparticles Using *Phyllanthus* Plant Extracts and Their Antimicrobial and Anticancer Applications

**DOI:** 10.3390/nano13192616

**Published:** 2023-09-22

**Authors:** Maxwell Thatyana, Nondumiso P. Dube, Douglas Kemboi, Amanda-Lee E. Manicum, Ntebogeng S. Mokgalaka-Fleischmann, Jacqueline V. Tembu

**Affiliations:** 1 Department of Chemistry, Tshwane University of Technology, Private Bag X680, Arcadia, Pretoria 0001, South Africa; thatyana.maxwell@gmail.com (M.T.); dubendumi@gmail.com (N.P.D.); kemboidouglas01@gmail.com (D.K.); manicumae@tut.ac.za (A.-L.E.M.); 2 Department of Chemistry, University of Kabianga, Kericho 2030, Kenya; 3 Mamelodi Campus, University of Pretoria, Private Bag X20, Hatfield, Pretoria 0028, South Africa; ntebogeng.mokgalaka-fleischmann@up.ac.za

**Keywords:** phytonanoparticles, antimicrobial, anticancer, antifungal, phytochemicals

## Abstract

Nanoparticles and nanotechnology developments continue to advance the livelihood of humankind. However, health challenges due to microorganisms and cancerous cells continue to threaten many people’s lives globally. Therefore, new technological interventions are of great importance. The phytochemicals present in medicinal plants are suggested as biocompatible, cost-effective, and regenerative sources that can be utilized for the green synthesis of nanoparticles. Different plant extracts with various phytochemical constituents can form nanoparticles with specific shapes, sizes, and optical properties. This review focuses on advances in green nanotechnology and provides details on reliable synthetic routes toward medically and biocompatible relevant metallic nanoparticles. We cover a wide range of applications that use phytonanoparticles with an in-depth look at what makes these materials interesting. The study also provides details of the literature on the interventions made in phytonanotechnology for the production of plant-mediated synthesis and capped metallic nanoparticles and their applications in various industries. It was observed that a variety of plants have been well studied, and detailed findings have been reported; however, the study of *Phyllanthus* is still in its early stages, and more needs to be uncovered.

## 1. Introduction

Nanotechnology is an emerging multidisciplinary research field described as the engineering, science, and technology of designing, fabricating, characterizing, and applying systems, structures, and devices at the nanoscale, typically in the range of 1 to 100 nm [1,2]. In the last decade, nanotechnology has made significant progress and shown great potential for application in the fields of physics, medicine, agriculture, biology, chemistry, and electronics [3,4,5]. Moreover, the integration of biotechnology and nanotechnology provides an environmentally friendly and green technology for the production, characterization, and application of nanomaterials [2,6]. Nanomaterials, especially metallic nanoparticles, have attracted great research interest due to their fascinating and unique optical, mechanical, and chemical properties related to their large surface-to-volume ratios [7,8].

**Figure 1:**
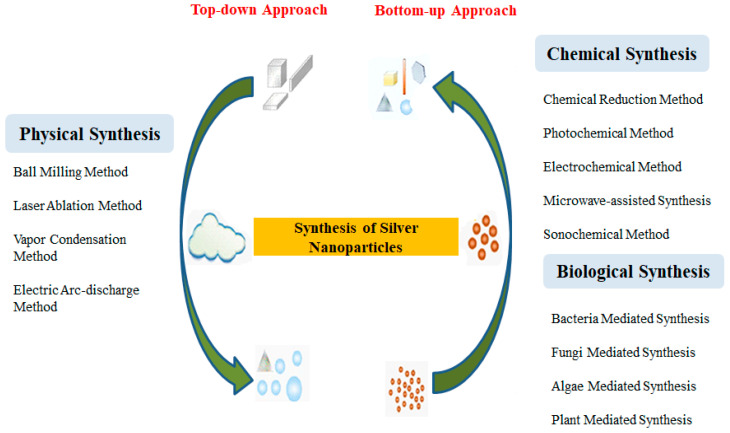
Silver nanoparticle synthesis: a bottom-up and top-down approach, i.e., physical synthesis method, chemical synthesis method, and biological synthesis method, separately. The bottom-up approach refers to the growth of complex clusters and the synthesis of nanoparticles from molecular components. In contrast, the top-down approach refers to the formation of metal nanoparticles from bulk materials (adapted from [14]).

Nanoparticles can be formulated by various methods, including physicochemical methods [9]. However, the disadvantages of physicochemical methods are mainly the handling of toxic chemicals, the need for high temperatures and pressures, and the generation of harmful toxic waste products [10,11]. Due to the aforementioned drawbacks, there is a need for less harmful, alternative methods for nanoparticle production. Consequently, there is an increasing demand for nontoxic, environmentally friendly, and cost-effective methods to produce metallic nanoparticles (MNPs) for their potential pharmaceutical and biomedical applications [12,13]. Figure 1 illustrates the different synthesis methods for the production of nanoparticles.

Green chemistry and green nanotechnology (also termed green synthesis modalities) utilize environmentally benign solvents and nontoxic precursor materials for the synthesis of nanomaterials. These processes are aimed at the elimination and/or reduction of toxic byproducts in reaction media that could harm our environment [14,15,16,17]. The remarkable advantage of green nanotechnology is the utilization of plant-derived phytochemical constituents, which serve as stabilizing, capping, and reducing agents for the transformation of metal ions to their metallic nanoparticle form [18].

The emergence of green synthesis of MNPs is a pioneering achievement in the field of nanobiotechnology. Therefore, the use of biological and natural resources such as plants [19,20], microorganisms [21,22,23,24,25], and algae [26,27] for the synthesis of MNPs has immense potential. Plant extracts have the added advantage of requiring less time to reduce metal ions [28]. This is because phytonanofabrication does not require the establishment of cell cultures and does not require long incubation times or high temperatures [29]. The rapid reduction of metal ions is due to the ability of plant constituents (functional groups) to donate electrons to metal ion complexes [30,31]. The synthesis of nanoparticles using green chemistry is the main reason that natural plant extracts are being considered for nanoparticle synthesis. The main advantage of green chemistry is that it allows the selection of environmentally friendly reducing agents, nontoxic materials for stabilization, and solvents [21,32]. A variety of compounds, such as amines, amides, alkaloids, flavonoids, phenols, terpenoids, proteins, and pigments, are present in plant extracts [23]. The aforementioned phytochemical constituents help in the stabilization and reduction of metal ions during the green synthesis of nanoparticles [29]. Therefore, this review aims to highlight the use of plant extracts from the genus *Phyllanthus* in the green synthesis of metallic nanoparticles and their potential applications in pharmaceutical and biomedical research. 

## 2. Rationale

The utilization of green synthesis methods for the synthesis of metal nanoparticles (MNPs) has gained significant attention in recent years due to their eco-friendliness and sustainable nature [33]. Plant extracts, particularly those derived from the *Phyllanthus* genus, have shown great potential for the synthesis of MNPs due to their rich phytochemical composition. This rationale aims to explore the reasons behind utilizing *Phyllanthus* genus plant extracts for the synthesis of MNPs and their biomedical applications. The *Phyllanthus* genus comprises a wide variety of plant species that are abundantly available in various regions globally [34]. These plants are commonly found in tropical and subtropical zones, and their accessibility makes them an attractive choice for NPs synthesis [34]. The relative ease of acquiring *Phyllanthus* genus plant extracts ensures a consistent and reliable source for NP synthesis. 

*Phyllanthus* genus plants are known to possess a diverse array of secondary metabolites, including polyphenols, flavonoids, alkaloids, terpenoids, and organic acids. These bioactive compounds have strong reducing, capping, and stabilizing potential, making them effective agents for the synthesis of MNPs. These phytochemicals exhibit strong reducing properties that facilitate the reduction of metal ions into NPs [34]. Additionally, the functional groups present in the extracts act as capping agents, imparting stability to the resulting NPs and preventing agglomeration. The synergistic effect of various phytochemicals present in *Phyllanthus* genus plant extracts enhances the efficiency and control over MNP synthesis. Different phytochemical constituents may contribute to distinct aspects of NP formation such as size, shape, and stability. The combined action of these compounds ensures the production of highly stable MNPs and improved performance in various applications. In contrast, the selection of *Phyllanthus* genus plant extracts as green agents for MNPs synthesis is well justified. Their abundance, phytochemical composition, reduction and stabilization mechanisms, synergistic effects, environmental benefits, biocompatibility, and cost-effectiveness render *Phyllanthus* genus plants highly suitable for the synthesis of MNPs. Exploring the vast potential of *Phyllanthus* genus plant extracts in nanotechnology applications contributes to sustainable and eco-friendly advancements in this field. This review highlights the benefits of *Phyllanthus* genus plant extracts in nanotechnology and their biodiverse applications in biomedical applications with a focus on antimicrobial and anticancer applications. 

## 3. Review Methods

This review incorporates entries from the scientific literature. Data for this review were collected from databases such as Google Scholar, Springer, Elsevier, PubMed, ResearchGate, MDPI, and Hindawi from 2012 to 2021 for retrieving information on pertinent keywords like green synthesis, green nanotechnology, phytonanoparticles, genus *Phyllanthus*, and phytonanotechnology. Publications and scientific articles were selected from reputable journals and sorted out to extract scholarly information on the green synthesis of metallic nanoparticles from the genus *Phyllanthus*. Moreover, this review consists of the first research report on the use of the *Phyllanthae* genus for the synthesis of various plant-based metallic nanoparticles and their applications in a wide spectrum of industries.

## 4. Common Techniques and Characterization of NPs for Surface Chemistry Data Collection

In general, nanoparticles are characterized by their size, surface area, shape, and dispersity [29,35]. These properties are critical for many applications of nanoparticles. The most common techniques for characterizing nanoparticles are ultraviolet-visible spectroscopy (UV-vis), scanning electron microscopy (SEM), transmission electron microscopy (TEM), dynamic light scattering (DLS), Fourier transform infrared spectroscopy (FTIR), energy dispersive spectroscopy (EDS), and powder X-ray diffraction spectroscopy (XRD) [35,36,37].

UV-visible spectroscopy is the most commonly used technique [38]. Various metal nanoparticles in the size range of 2–100 nm are generally characterized in the wavelength range of 300–800 nm [39]. Spectrophotometric absorption bands in the wavelength ranges of 400–450 nm [40] and 500–550 nm [40] are assigned to silver and gold nanoparticles, respectively. Electron microscopy is another commonly used characterization technique [41]. Transmission electron microscopy and scanning electron microscopy are used for morphological characterization at nanometer and micrometer scales [42], but TEM has a 1000 times higher resolution than SEM [43]. Dynamic light scattering is used to characterize the size distribution and surface charge of particles suspended in a liquid solution [44].

FTIR spectroscopy is used to characterize the surface chemistry of the particles [39], detecting organic functional groups such as hydroxyl, carbonyl, etc., bound to the surface of the nanoparticles and chemical residues. The elemental composition of the metal nanoparticles is usually determined using energy-dispersive spectroscopy [45]. Similarly, phase identification of the crystal structure of the particles is performed using XRD [46]. X-rays penetrate the nanoparticles, and the resulting diffraction patterns are compared with reference standards to obtain the structural information of the nanoparticle [47].

Different scientific tools can be utilized to characterize the surface chemistry, morphology, and shape of the synthesized nanoparticles. As mentioned earlier, UV-visible spectroscopy can confirm the bioreduction of nanoparticles through the acquisition of surface plasmon resonance peaks. UV-visible spectroscopy can also provide crucial information about the width, shape, band, size, and possible aggregation state of nanoparticles [48]. Techniques such as AFM, SEM, TEM, and STM analysis can be utilized to study the NPs’ topology, morphology, size, surface roughness, shape, and texture. XRD is useful for the study of the crystal structure of the synthesized NPs [49,50]. Another useful technique is the DLS, which can be used to determine the size and aggregation nature of NPs. The functional groups and biomolecules of the stabilizing and reducing agents present on the NP’s surface can be identified with FTIR spectroscopy [51], respectively. Additionally, the purity and elemental composition of NPs can be studied with EDAX analysis [52]. Various other techniques can be used to verify the desired NPs; however, the listed tools are the basic ones and can offer useful information about the achieved synthetic nanomaterials.

## 5. Factors That Influence the Synthesis of Nanoparticles from Plants

There are various factors influencing the synthesis of nanoparticles from plant extracts. For example, the plant extract concentration needs to be optimized [53]. A correct quantity of plant extract enhances the size and shape of nanoparticles and increases their production [54]. Another critical factor is the reaction temperature, which directly influences the size and shape of nanoparticles. In addition, the temperature of the reaction directly affects the reaction rate, which affects nanoparticle characteristics. Consequently, one can customize the desired properties, including size, shape, growth, and particle distribution, by simply altering the temperature of the reaction [54,55,56].

In addition, the pH of the solution influences the synthesis rate, size, and shape of NPs [57,58]. The nucleation centers increase with the increase in pH, transforming the metal ions into their solid metallic state. The solution’s pH enhances the reaction rate by affecting the activity of the functional groups present in the plant extract. Singh and coworkers (2010) [59] postulated that at lower pH, gold (Au) tends to aggregate to form bigger-sized NPs; however, more carbonyl and hydroxyl groups are available for Au binding at higher pH. Furthermore, in the synthesis of silver NPs (Ag NPs) from silver nitrate, with glucose used as a reducing agent, sodium hydroxide as an accelerator, and starch as a stabilizer, different surface plasmon resonance (SPR) was observed at different pH values [59].

Another crucial factor is the reaction time. In a study by Dwivedi and Gopal (2010) [60], they reported an increase in the sharpness of UV-vis absorption bands with an increase in contact time. They observed the formation of nanoparticles within 15 min of the reaction, and the synthesis rate increased to 2 h, respectively [60]. Furthermore, Dubey and coworkers (2010) [58] observed that the formation of Ag and AuNPs started within 10 min of the reaction time. They further observed that an increase in contact time was responsible for sharpening the absorption peaks for both Ag and AuNPs [58].

## 6. The Role of Plants in the Synthesis of Nanoparticles

Metallic nanoparticles have a wide range of commercial applications, which are constantly expanding. Many academics now embrace the use of biological synthesis since it is secure, economical, and ecologically beneficial [61]. Plants have been extensively researched in recent years as sources for the production of metallic nanoparticles from their inorganic metal ions [54]. The phytochemicals found in plants are also essential for the reduction of metal ions. Reduced maintenance and waste disposal costs, less toxic waste production, positive impacts on treatments and the action of the extracts as both reducing and stabilizing agents are benefits of using plant extracts in the synthesis of metal NPs [62].

Plant extract is combined with a metal precursor solution under various reaction conditions to create nanoparticles [63]. The rate of nanoparticle formation, as well as the yield and stability of the nanoparticles, are administered while taking into account the reaction parameters governing the conditions of the plant extract, such as (i) the metal salt concentration, (ii) the phytochemical concentration, (iii) the solution pH and temperature, and (iv) the type of phytochemical [64]. Also, compared to bacteria and fungi, which require longer incubation times, the phytochemicals found in plant extract have a higher tendency to reduce metal ions in a shorter amount of time [65]. As a result, plant extracts are thought to be great candidates for the creation of metal and metal oxide nanoparticles [66]. Plants have various concentrations of phytochemicals, which is another crucial factor in the creation of nanoparticles [67,68]. Sugars, carboxylic acids, flavones, terpenoids, amides, ketones, and aldehydes are the primary phytochemicals found in plants that are involved in the bioreduction of nanoparticles [67].

**Figure 2:**
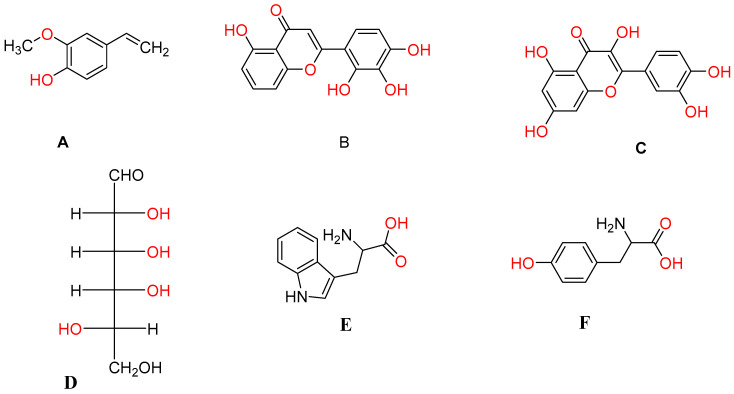
Types of plant metabolites involved in the synthesis of metal nanoparticles: (**A**): terpenoids (eugenol); (**B**,**C**): flavonoids (luteolin, quercetin); (**D**): a hexose with the open-chain form; (**E**,**F**): amino acids (tryptophan (**E**) and tyrosine (**F**)) (adapted from [67]).

The numerous plant metabolites stated above are crucial to the bioreduction of metal ions into nanoparticles, as was hinted at above. The primary substances capable of reducing metal ions are shown in Figure 2 below.

Alkaloids are basic nitrogenous compounds with pharmacological and definite physiological activity. Alkaloids are among the diverse phytochemicals of the *Phyllanthus* genus; at least 12 *Phyllanthus* species were found to contain about 22 different alkaloids of the securinene/norsecurinine type. These have a unique tricyclic skeleton with an α,β-unsaturated-γ-lactose ring. Securinine is arguably the first alkaloid found in many *Phyllanthus* species. Moreover, phyllanthine was the most common alkaloid and was isolated from three different *phyllanthus* species [62]. 

Terpenoids are a diverse class of organic polymers synthesized in plants formed from isoprene units and they display strong antioxidant activity. An initial study by Shankar et al. (2003) revealed that terpenoids play a pivotal role in transforming silver ions into nanoparticles in reactions using extracts from geranium leaves [62]. Eugenol, the main terpenoid, was found to play the principal role in the bioreduction of AgNO_3_ and HAuCl_4_ to yield nanoparticles [69]. According to Singh and coworkers (2010), the FTIR spectroscopic data suggested that dissociation of the eugenol proton (-OH) group results in the formation of a resonance structure capable of further oxidation. This process is accompanied by the active reduction of metal ions, followed by nanoparticle formation [59].

**Scheme 1:**
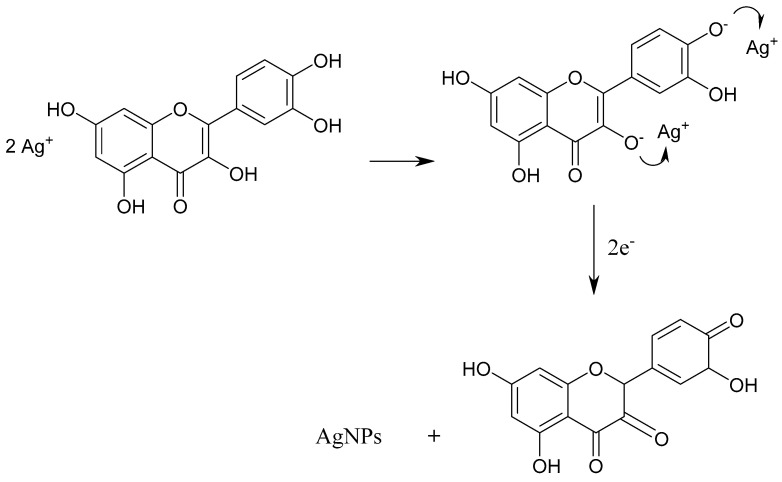
Reduction mechanisms of Ag ions to AgNPs by quercetin molecule (adapted from [71]).

**Scheme 2:**
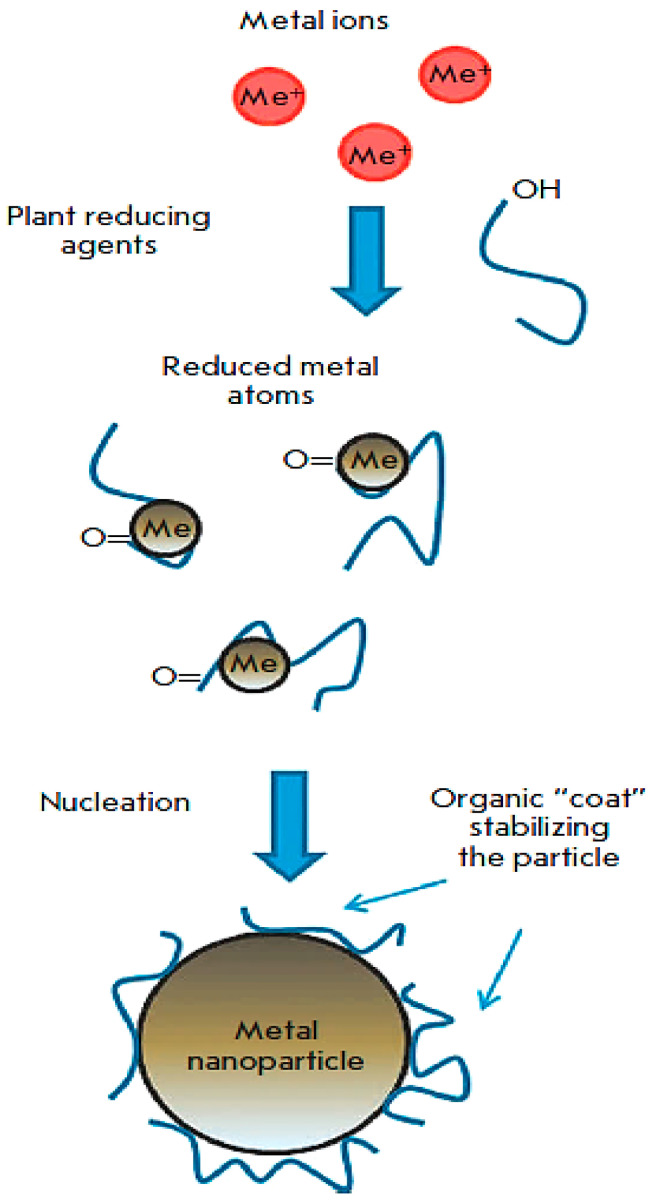
A representation of the plant-mediated synthesis of metal nanoparticles (adapted from [67]).

Flavonoids are polyphenolic compounds that comprise several classes such as flavanones, anthocyanins, flavonols, flavones, isoflavonoids, and chalcones, which actively reduce chelated metal ions into nanoparticles [70]. Various functional groups on flavonoids are capable of transforming metal ions into nanoparticles. The study by Ahmad and coworkers (2010) [69] postulated that the tautomeric transformations of flavonoids from the enol-form to the keto-form may release a reactive hydrogen atom capable of reducing metal ions to nanoparticles. Moreover, the conversion of ketones to carboxylic acids through the internal mechanism in flavonoids is likely involved in Au^3+^ ion reduction. Interestingly, some flavonoids can chelate metal ions with their π-electrons or carbonyl groups, respectively [69]. Scheme 1 shows the mechanism of the photochemical reduction of Ag ions to form nanoparticles, and Scheme 2 represents metal nanoparticle formation with plant extracts as reducing and stabilizing agents. 

Scheme 2 shows metal ions binding to the stabilizing agents and reducing plant metabolites, and the ions are reduced to metal atoms. The resulting complexes interact with similar complexes, forming a small metal nanoparticle. This is followed by the growth and coalescence of separate small nanoparticles into larger ones that occur through the coarsening process. This process continues until the particles assume a stable shape and size for nanoparticles. Below is the scheme of nanoparticle formation with plant extract [72].

### 6.1. Green Synthesis Methods of MNPs Using Phyllanthus Plant Extracts

**Figure 3:**
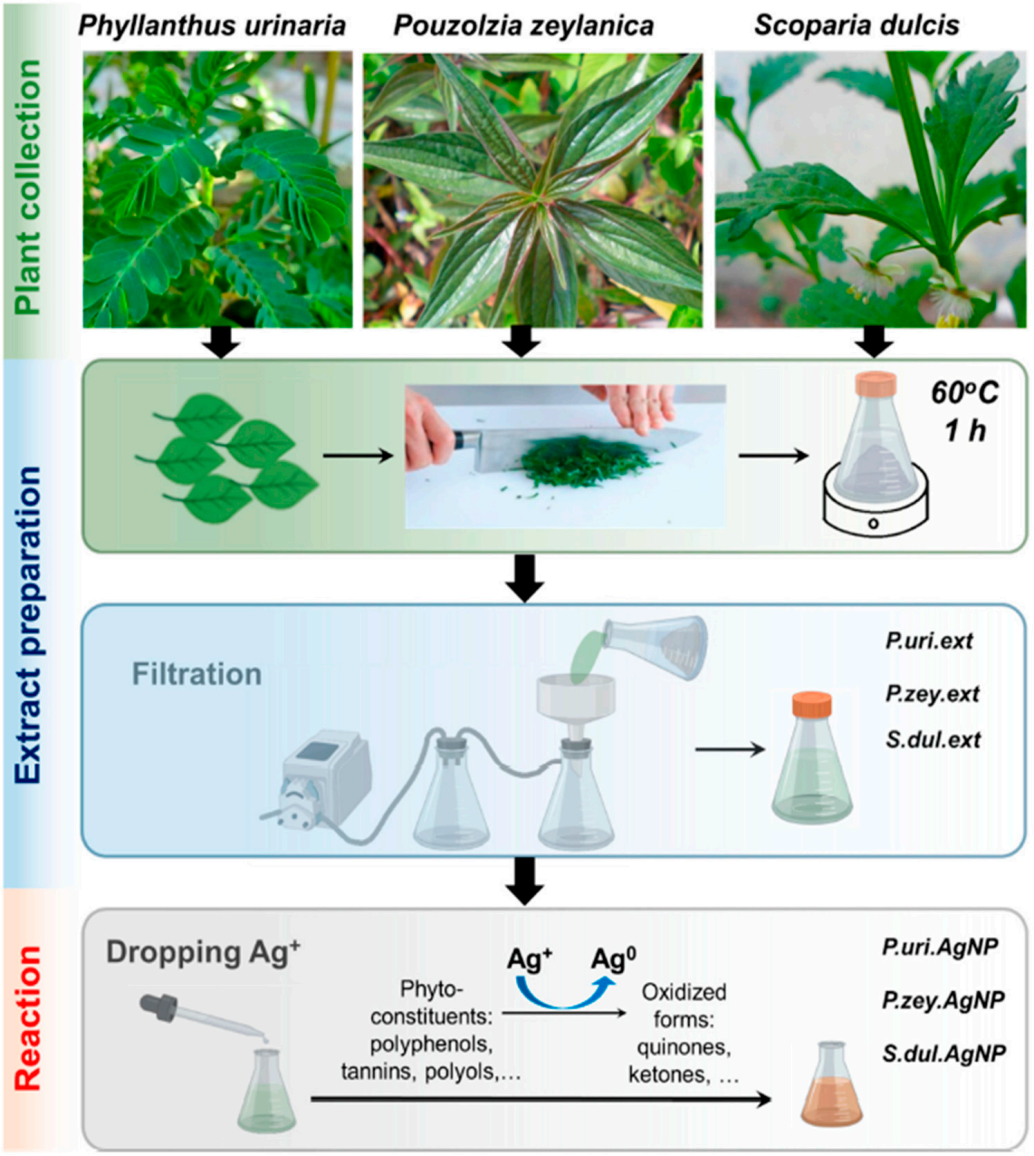
Synthesis of metal nanoparticles using plant extracts (adapted from [73]).

This section discusses the detailed synthesis of MNPs using the *Phyllanthus* plant extracts. Moreover, it also highlights detailed methods for obtaining reducing plant extract materials used in the synthesis from different *Phyllanthus* plants. As an illustration, Figure 3 demonstrates the synthesis route for MNPs from various plant extract. As observed from Figure 3, the plant materials (leaves in this case) are first chopped into smaller pieces and transferred into an extraction container. Then the plants in a solvent are heated at 60 °C for 1 h; the resulting solution is then filtered to collect the plant extracts. After filtration, the extracts are then used to synthesize Ag nanoparticles by adding silver nitrate into the plant extracts to form AgNPs. Figure 3 below illustrates the synthesis route for MNPs formation using plant extracts. 

#### 6.1.1. Synthesis of AgNPs Using *P. emblica* Fruit Extracts by [74]

The study by Musam and co-workers (2019) reported the synthesis of AgNPs using *P. emblica* fruit extracts [74]. The fresh fruit extracts of *P. emblica* plants were prepared by following a procedure reported by [75]. Briefly, the fresh fruits were cleaned with sterilized double-deionized water and then chopped into small pieces, after which the seeds were removed. The sliced fruits were then finely macerated using a blender through sterile ddH_2_O to obtain 10% (*w*/*v*) fruit broth. The resulting extracts were then passed through a muslin cloth and then filtered using Whatman No. 1 filter paper and kept at 4 °C for later use. Furthermore, the NPs were synthesized as follows: In a 100 mL aqueous solution of AgNO_3_ (1 mM), various concentrations of aqueous fruit extracts (2.5, 5, 10, and 15 mL) were added and heated at 65 °C for 20 min and then kept at room temperature under dark conditions. The resulting NPs were then confirmed via color change and UV-Vis measurements. 

**Figure 4:**
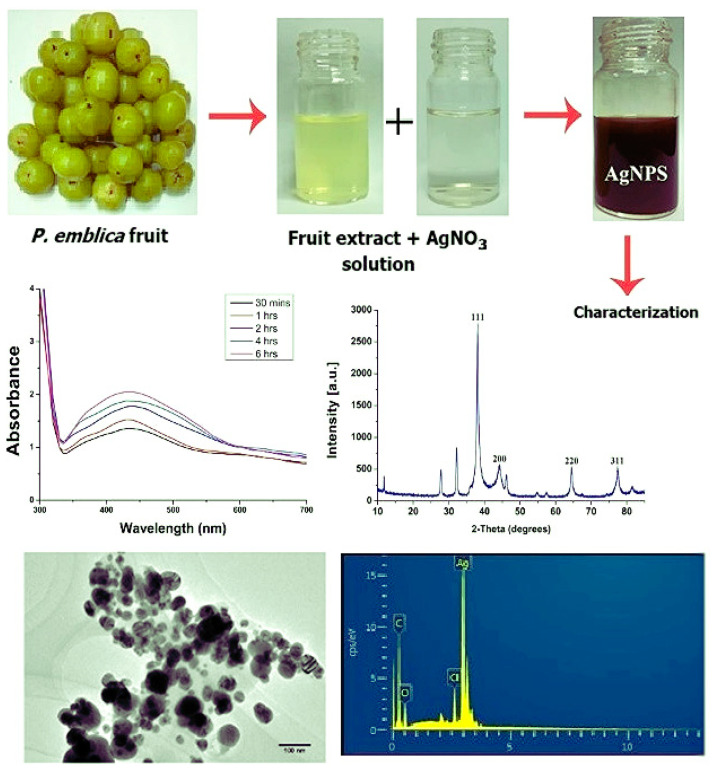
The synthesis and characterization of MNPs using various aqueous fruit extracts were edited for this manuscript (adapted from [74]).

For example, Figure 4 below demonstrates the synthesis of AgNPs from various fruit extracts. The NPs are further confirmed with various scientific techniques. As illustrated below, visual analysis was carried out using UV-vis to confirm surface plasmon of the NPs, FTIR to study the surface chemistry, FESEM to study the NP topology, TEM to confirm the NP size, Zeta potential to study the surface charge of the NPs, XRD to study the crystallinity and diffraction patterns of the NPs, AFM to study the surface roughness and topology, and lastly, EDX to confirm the elemental composition of the NPs. This is to demonstrate that after the synthesis of MNPs, different scientific techniques were employed to acquire more information about the synthesized MNPs. As a result, it can be confirmed from the acquired data in comparison to the scientific databases that the NPs formed are the desired materials under investigation. Figure 4 below is the illustration of the synthesis of MNPs and their characterization.

#### 6.1.2. Synthesis of AgNPs Using *P. emblica* Methanol Fruit Extracts by [76] 

Another study by Dhar and co-workers (2021) [76] reported the synthesis of AgNPs using *P. emblica* fruit extracts. The fruit extracts were prepared as follows: Fresh fruits of 80 g were cleaned carefully with DIH_2_O sliced into small pieces (3–5 mm in size) and dried at a specified temperature. Then, 20 g of chopped and dried fruit was placed into a 100 mL aqueous solution of 70% (*v*/*v*) methanol. The solution was then boiled at 60 °C while stirring for 30 min. The resulting solution was cooled at room temperature and filtered twice through Whatman filter paper (Qualitative, Φ18.5 cm) and stored at 4 °C for later use. NP synthesis was carried out as follows: In a reaction vial, 20 mL of fruit extracts of *P. emblica* were mixed with 180 mL of 1 mM solution of AgNO_3_. The mixture was stirred continuously at 65 °C for 1 h, after which a color change from light green to light brown was observed. The solution was then kept under incubation for 24 h and the color changed from light brown to dark brown, indicating the formation of AgNPs. The resulting NPs were confirmed with UV-Vis.

#### 6.1.3. Synthesis of FeNPs Using *P. niruri* Plant Leaf Extracts by [72]

A study conducted by Kumar and Prem (2018) [72] reported the synthesis of FeNPs using the leaf extracts of *P. niruni*. The leaf extracts were prepared by cleaning under running water followed by DIH_2_O. About 25 g of leaves were boiled in 100 mL of DIH_2_O for 2 h. The extracts were then filtered through a Whatman No. 1 filter paper and cooled at room temperature. The resulting extract was stored for later use. Before the synthesis, a mixture of ammonium iron(III) sulfate decahydrate 9.6516 g was dissolved in 100 mL of DIH_2_O and 3.9213 g of ammonium iron(II) sulfate hexahydrate in 100 mL separately. Then, 5 mL of both solutions were mixed to have iron-salt mixture ratios of (1:2; 1:3; 1:5; and 2:3). The synthesis of FeNPs was performed by adding leaf extracts (1.2 mL) to 10 mL of iron salt mixtures, and the reactions were stirred for 30 min at 30 °C, after which the reddish-yellow solution turned to black-grey. The resulting FeNPs were separated with a magnet and confirmed with UV-Vis. 

## 7. Nanoparticles Stability Test

The optimum in vivo and long-term stability of NPs is imperative for the use of NPs in biological applications. An in vitro stability study is performed on metal NPs by mixing the nanoparticles with biological media such as human serum albumin, cysteine, bovine serum albumin, and histidine in various pH solution ranges to mimic in vivo conditions [77]. The surface plasmon resonance peaks from NPs were monitored to see the stability of NPs mixed with various solutions. UV-visible was used to monitor the absorption spectra of the NPs to determine the stability of the NPs. In other studies by Kattumuri and co-workers (2007) [75], the introduction of an electrolyte method was used to study the stability of AuNPs produced using gum Arabic. Moreover, zeta-potentiometry was also used to provide the stability information of AuNPs because AuNPs with low surface charge tend to agglomerate, and over time the NPs gained a stable state.

## 8. Phytochemical Constituents with Metal Ion Reduction Capacity

Plant extracts, which are also known as secondary metabolites, contain a variety of phytochemicals that help to reduce and stabilize metal ions in nanoparticles (NPs), as was briefly mentioned in the section above [76,78]. Enzymes, polysaccharides, organic acids, proteins, amino acids, vitamins, etc., are only a few examples of bioactive phytochemicals. The role of a few secondary metabolites that are common in plants as reducing agents for metal ions is addressed below.

### 8.1. Sugars

It has been proven that plant sugar extracts can cause metal nanoparticles to become stable. The capping abilities of sugar extracts were recently shown by Fillipo and co-workers (2010) [78]; Pattnaik and co-workers (2023) [79] depending on the presence of non-soluble carbohydrates like starch. In their 2011 study, Shervani and Yamamoto [80] used the stabilizing and structure-directing agents’ soluble starch (polysaccharide) and *β*-α-glucose (monosaccharide) in the synthesis of spherical NPs and nanowires of gold and silver. The sugar concentration prevented the formation of NP agglomerates, which led to the discovery that the produced NPs were relatively stable. The C-6 position of the sugar was discovered to have oxidized to a carboxylic acid in contact with auric acid, producing AuNPs with glucoside substituents at the anomeric carbon center [79]. By anomerizing the released aldehyde and then oxidizing it, auric acid was reduced. Similar findings were reported by [67], who found that phenyl *β*-α-glucoside provided the maximum yield of mono-disperse cylindrical AuNPs in his investigation using ^13^C nuclear magnetic resonance (NMR) measurements.

### 8.2. Alkaloids

The indolizidine alkaloids, ergoline alkaloids, benzenoids, and phenolic chemicals identified in the extracts of *I. pes-caprae* roots demonstrated the stability and reduction capability of Ag ions to AgNPs in a study conducted by Subha and coworkers (2015) [81]. The team reported that the FTIR analysis of this extract in combination with silver salt revealed peaks at 1660, 1043, and 635 cm^−1^ that were attributed to the protein amide’s C=O stretching vibrations, cyclic alcohols’ C-OH stretch vibrations, and aromatic C-H vibrations that were attributed to the presence of free quinones derived from polyphenolic compounds in the extract [82]. The group called attention to the fact that only a small number of academic works discuss how alkaloids contribute to the conversion of metal ions into nanoparticles. More investigations are needed in this area in the future. Even though the scope of this research does not include noticeable reports on the synthesis of metal nanoparticles using alkaloids, other plant extracts have demonstrated the potential of alkaloids in the synthesis of MNPs. A study by Almadiy and Nenaah (2018) [83] has demonstrated the synthesis of AgNPs using potato steroidal alkaloids and their activity against phytopathogenic fungi. This goes on to illustrate the need for investigations of alkaloids of the genus Phyllanthus for the synthesis of MNPs. Furthermore, Almadiy and co-workers (2018) [83] also demonstrated the synthesis of AgNPs using harmala alkaloids. They also investigated NPs for their insecticidal activity against khapra beetles. Again, this indicates that alkaloids are capable of reducing metal ions to their NP counterparts. 

### 8.3. Flavonoids

**Scheme 3:**
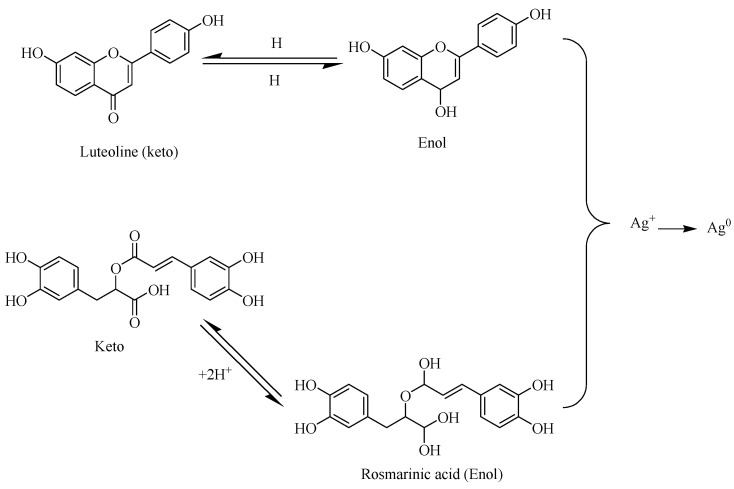
Keto-enol tautomerism and its effects on the synthesis of AgNPs (adapted from [84]).

Many families of polyphenolic chemicals known as flavonoids avidly chelate and decrease metal ions to their NP form [72]. Due to their availability of important functional groups like carbonyl and hydroxyl moieties, metal ions can be reduced [84]. Consequently, it was demonstrated that a *Rumex dentatus* aqueous water extract with a high phenolic and flavonoid content allowed for the simple reduction of Ag^+^ ions to Ag^0^ [71]. According to a study by [85], the release of reactive hydrogen atoms from flavonoids during their transformation into keto-enol tautomers allows metallic ions to be reduced to their nanoparticle form. For instance, it was claimed that flavonoids and rosmarinic acid from *Ocimum basilicum* extract generate the keto-enol mechanism, which is essential for the development of NPs and is seen in the picture below [85]. Furthermore, the internal conversion of ketones to carboxylic acids in flavonoids is probably the cause of the reduction of Au^3+^ metal ions. Contrarily, flavonoids readily bind to the surfaces of developing NPs because they chelate metal ions such as Cu^2+^, Zn^2+^, Fe^2+^, Fe^3+^, Pb^2+^, Cr^3+^, and Co^2+^. This means that they have an impact on early nucleation, limit aggregation, and facilitate the bioreduction of metal ions [86]. As seen in Scheme 3 below, the hydrogen reacts with the Ag^+^ ion resulting in the keto-enol tautomerism mechanism of reaction for the reduction of Ag^+^ to Ag^0^ form. This mechanism has been shown to be the mode of AgNPs formation with phytochemicals as reducing and stabilizing agents. 

### 8.4. Terpenoids

**Scheme 4:**
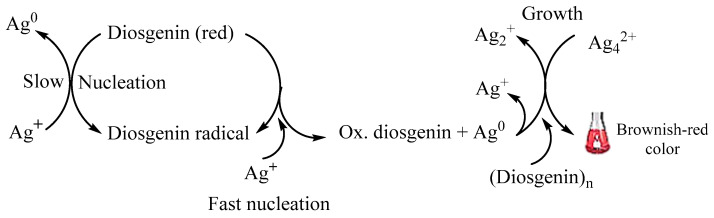
Proposed mechanism for AgNP formation by reduction with steroidal saponin diosgenin (adapted from [86]).

In a study by [87], they discovered that terpenoids are frequently linked to the formation of metallic NPs based on FTIR results. According to a study by Shankar and colleagues (2003) [62], the presence of terpenoids in leaves was responsible for the creation of AuNPs when geranium leaves and chloroaurate ions reacted. Also, the group came to a similar conclusion: the high content of eugenol in *Cinnamomum zeylanisum* (cinnamon) extracts was substantially responsible for the bioreduction of HAuCl_4_ and AgNO_3_ by the extracts. According to earlier research, deprotonation of the hydroxyl group in eugenol results in an anion that is further oxidized by metal ions, reducing the ions to metallic NPs [88]. Also, it was shown that the steroidal saponin diosgenin served as a capping and reducing agent during the creation of AgNPs; Scheme 4 below elaborates on the suggested reduction mechanism [78] where diosgenin red reacts with Ag^+^ ions to form the resulting AgNPs.

## 9. The Genus *Phyllanthus* and Its Phytochemical Constituents

The *Phyllanthaceae*, a large family of flowering plants with around 1301 species, includes the genus *Phyllanthus*. It is extensively dispersed in the tropical and subtropical regions of Africa, America, Asia, and Australia [89,90]. The most significant historically employed species of the *Phyllanthaceae* family for the treatment of various human maladies are the *Phyllanthus Cicca* and *P*. *Kirganelia* [36]. *Phyllanthus* plants contain a variety of phytochemical components that are significant in pharmacology. Among these phytochemicals are terpenoids, alkaloids, and polyphenolic substances such as phenolic acids, flavonoids, coumarins, lignins, stilbenes, and anthocyanins, among others [91].

Husnunnisa and colleagues (2022) [92] tabulated the phytochemical components of the *Phyllanthus* plant family, their pharmaceutical applications, and the biological activities of several *Phyllanthus* species [93]. The pharmacological and biological activity of chemical compounds isolated from diverse *Phyllanthus* plant species against common diseases were listed in a review study by Calixto and colleagues (1998) [93]. These chemical characteristics make the *Phyllanthus* genus a fascinating group of plants that should be researched further for possible medication development and future biological applications [91].

**Figure 5:**
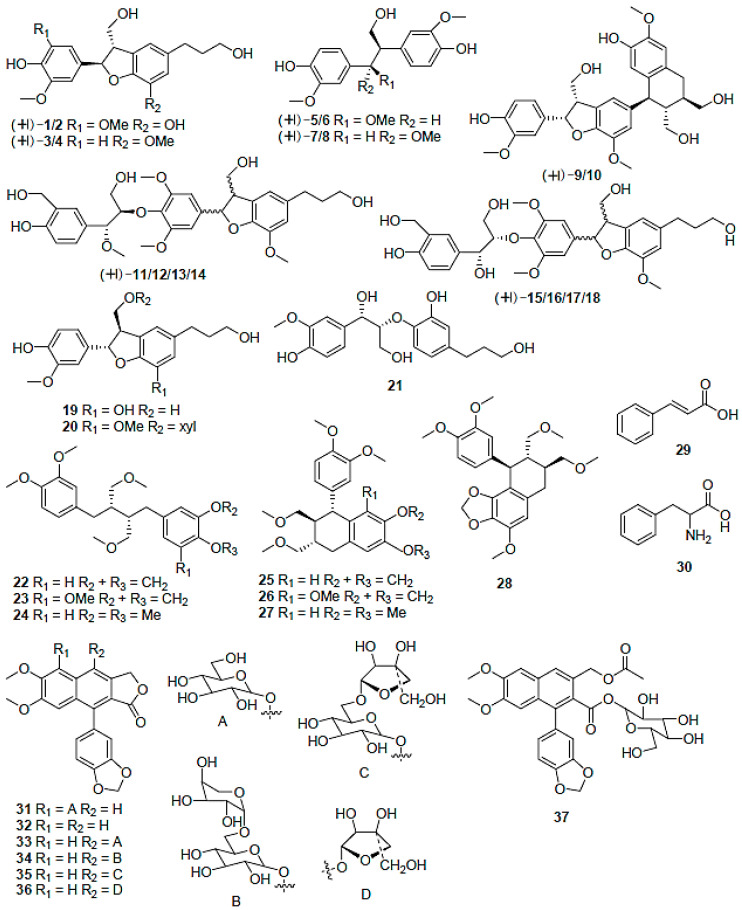
Phenylpropanoids from various *Phyllanthus* species (adapted from [89]).

**Figure 6:**
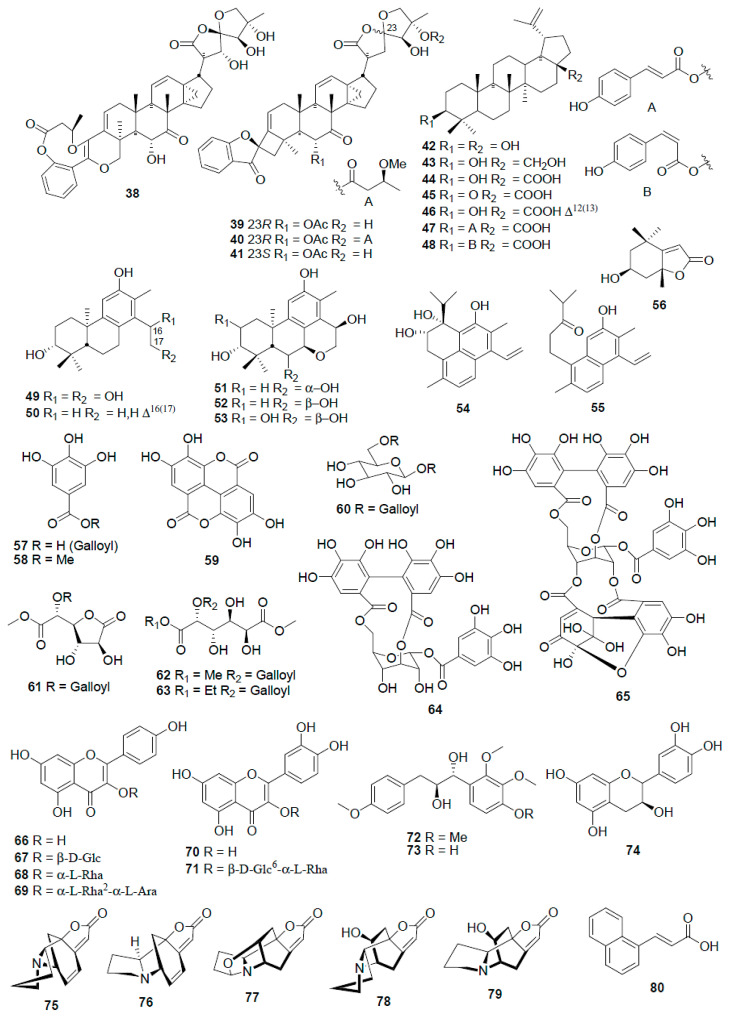
Flavonoids, alkaloids, and other compounds from various *Phyllanthus* species (adapted from [89]).

Indeed, many plants of *Phyllanthus* species have long been used as traditional medicines in the treatment of various diseases, mainly kidney, urinary bladder disturbances, intestinal infections, diabetes, and hepatitis B. The medicinal value of these plants, however, is influenced by some chemical substances that produce definite physiological actions in the human body. Given the healing properties of species in the genus, much interest has been focused on the chemical components of these plants. The preclinical and clinical studies carried out with the extracts and purified compounds from these species support most of their reported uses in folk medicine for the treatment of a wide variety of pathological conditions. The species in this genus have been reported to contain terpenes, alkaloids, lignans, flavonoids, and tannins with various kinds of bioactivities. The phytochemical constituents of the *Phyllathus* genus are summarized in Figure 5 and Figure 6.

In addition, phytochemical compounds play a vital role in MNP synthesis as reducing agents and stabilizers. Polyphenol compounds, proteins, and reducing sugars are the main phytochemical compounds that are responsible for the reduction, stability, and synthesis of MNPs [90]. Moreover, green synthesis is a method of synthesizing nanoparticles using natural materials that are environmentally friendly and safe. The use of green chemistry methods to synthesize NPs has attracted wide research interest due to its potential advantages [94], such as environmental friendliness, low energy consumption, cost-effectiveness, non-toxic, lack of pollution, and greater sustainability [94,95,96]. Lastly, NPs produced using green chemistry methods are relatively more stable and safer than those produced using traditional physicochemical methods [82,83]. 

## 10. Plant-Mediated Synthesis of Nanoparticles Using the Genus *Phyllanthus*

Nanoparticles in nature can be fabricated using two distinct approaches: the bottom-up method or the top-down approach (Figure 1) to obtain nanostructures with desired functionalities, shapes, and sizes [97]. The top-down approach requires diverse synthesis approaches like lithographic techniques, ball milling, sputtering, and etching to generate nanomaterials [98]. However, these techniques result in undesirable disadvantages like the production of hazardous byproducts, excessive energy use, chemicals harmful to the environment, etc. Furthermore, the bottom-up approach is the most widely used and efficient method for synthesizing nanoparticles, which involves aggressive reducing agents, volatile solvents, and capping agents. These two methods effectively produce pure and well-defined metallic nanoparticles, but their high production costs hinder their use [99]. 

The development of nanoparticles from plants has drawn tremendous attention and gained much importance in recent years owing to its eco-friendliness and simplicity [95]. In a review article by Gour and Jain (2019) [95], they elaborated on the synthesis of various metallic and oxide nanoparticles using different plants. Moreover, their study demonstrated various green synthetic methods of nanoparticle synthesis from enzymes, vitamins, microwave-assisted synthesis, bacteria and actinomycetes, yeast and fungi, algae, and plants, and their characterization and applications in pharmaceuticals [99]. Elsewhere, Yadi and coworkers (2018) [99] demonstrated the green synthesis of various nanoparticles, including Pd NPs, ZnO NPs, CuO NPs, CeO_2_ NPs, Ag NPs, and Au NPs (particle size not highlighted) from different plants [100]. Many studies have reported that plant extract-mediated metal NPs not only are biocompatible and non-toxic with normal human cells but also afford targeted drug delivery due to the localization of NPs in particular areas; they also exhibit anticancer, antimicrobial, and antiviral properties [101,102].

Table 1 below summarizes the green synthesis of Ag NPs, Au NPs, Cu NPs, Fe_2_O_3_ NPs, MgO, and Pt NPs from *Phyllanthus* plant extracts and their potential biological application against microorganisms. From the studies tabled below, it can be shown that phytonanoparticles hold great potential to serve as future bactericidal and fungicidal agents. However, most research by various groups did not carry out the minimum inhibitory concentration studies, which would eventually quantify their findings. Minimum inhibitory concentration studies are of paramount importance when studying drugs and/or materials to monitor their activity and also confirm microorganism resistance toward the studied materials. Therefore, the limitation of most of the researched work is that the groups performed screening (disc diffusion) studies only to report their work, which in our view does not give evidence of their activity.

However, the reported results indicate that even at lower concentrations (50 mg/mL to 200 mg/mL), the tested phytonanoparticles could enhance microbial cell death with a zone of inhibition of more than 12 mm. These findings could have propelled the research works to determine the MIC for the studied NPs. Furthermore, it can be observed that AgNPs dominate the most in terms of activity toward antimicrobial activity, with a MIC value of 10 µg/mL against most known pathogenic microorganisms. It is also observed from the research findings that the studied NPs are effective in reducing cell mortality even at very low concentrations (between 20 to 200 mg/mL), which is an ideal consideration of interest for their biological applications in the future over their synthetic counterparts. The reason for these great findings is that NPs can penetrate microbial cells and release reactive oxygen species (ROS), which promote apoptotic cell death. 

Similar observations can be made in Table 2. This is because a similar mechanism of activity takes place where NPs promote cell penetration and the release of ROS and cell damage. Additionally, AgNPs also dominate cell mortality. The NPs demonstrated strong activity against HeLa cell lines, with IC50 values of 15–50 µg/mL. Another interesting observation of these studies is that most of the reports showed that the NPs were not toxic toward normal cell lines, which is one of the attributes desired for a prominent drug and/or material. As a consequence, further studies in the field of phytonanotechnology need to be investigated to overcome the challenges reported about synthetic drugs’ counterparts. This suggests that there is great potential for natural product discovery with major efficacy on cancer cells while sparing normal cells. Therefore, more research emphases should be interrogated and significant findings will be made shortly that will improve the quality of life for many people globally. Phytonanotechnology holds all the desired solutions to human being’s known challenges today.

## 11. Nanoparticle Uptake and Interaction with Cell Mechanisms

A NP’s mode of cell penetration can be through ion exchange and cellular pores in the cell membrane and is dependent on the NP’s size. The uptake of NPs by cells is not solely dependent on membrane cell receptors but can also be through Van der Waals forces, electrostatic interaction, or steric interactions [122]. The size of the NP triggers different cellular effects based on the localization of the NPs in the cell. Some small metallic NPs at high concentrations are readily endocytosed by cellular vesicles. Furthermore, macropinocytosis and phagocytosis are carried out by neutrophils and macrophages [123]. Moreover, when protein-coated NPs interact with neutrophils and macrophages at the site of inflammation, the protein corona on the NP’s surface comes into contact with the cell surface receptors first [124]. The protein corona has serum proteins that act as ligands for receptors on the M2 macrophages, which then activate the anti-inflammatory M2 macrophages. These M2 macrophages play a pivotal role in NP uptake by cells [122].

In a study conducted by Binnemars-Postma and co-workers (2016), they found that in the presence of a serum protein, M2 macrophages exhibit rapid and high NP uptake compared to M1 macrophages. A phagocytosis gene array study on M1 and M2 cells exhibited an increase in the expression levels of receptors for complement factors (FCGR2B and CD36 receptors) and immunoglobulins in M2 macrophages when compared to M1. These observations led to the conclusion that M2-induced receptors bind to the protein corona [125]. This concludes that the adsorption of serum proteins (complement factors and immunoglobulin) enhances the uptake of NPs by M2 macrophages. Furthermore, neutrophils from extracellular traps (NETs) around M2 macrophages respond to endogenous stimuli like cholesterol or uric acid and exogenous stimuli like foreign particles or pathogenic microbes. The NET formation depends on receptor-interacting protein kinase-3 (RIPK-3) enzymes as well as reactive oxygen species (ROS) radicals [125]. ROS are highly reactive and unstable as they contain unpaired electrons in their outermost shells. They are formed by lipid peroxide formation, which causes cell membrane damage [126].

## 12. Application of Plant-Mediated Synthesized Nanoparticles

### Phytonanoparticles for Antimicrobial Activity

The green synthesis of nanoparticles using phytochemicals as mentioned before offers a wide range of advantages for their application in nanomedicine. A study conducted by Balachandar and coworkers (2019) [102] successfully demonstrated the synthesis of silver nanoparticles using stem extracts of *Phyllanthus pinnatus*. This group revealed that phytochemicals such as alkaloids, alcohols, saponins, terpenes, phenols, and proteins are responsible for the photosynthesis of AgNPs. The nanoparticles’ surface plasmon resonance (SPR) λ_max_ absorption peak was observed at 490 nm. The nanoparticles’ shape and surface morphology demonstrated that the nanoparticles were cuboidal and, to a lesser extent, spherical and triangular.

Furthermore, Balachanda and coworkers tested the synthesized nanoparticles for their antimicrobial efficacy against *Vibrio cholera*, *Shigella flexneri*, *Pseudomonas aeruginosa*, *Mycobacterium smegmatis*, *Proteus vulgaris*, and *Bacillus subtilis*. They found that all bacterial pathogens exhibited dose-dependent inhibition. The highest measured zone of inhibition was at 1.8 mm at a concentration of 40 µL. The study was somewhat inconclusive in that the group did not perform further studies to measure the minimum inhibitory concentration, giving concrete evidence of their antibacterial activity. However, it was highlighted that these studies would be carried out later [104].

A study conducted by Sivakumar and coworkers (2017) [127] also demonstrated the phytosynthesis of AgNPs from a leaf extract of *Phyllanthus urinaria* L. They speculated that polyols such as (flavones and catechins) might be involved in the reduction of silver ions to form AgNPs. In addition to that, the group also observed the disappearance of the C-O band at 1226 cm^−1^ in the IR spectra ascribed to the polyols. This corroborated the findings of other research groups [62,74,76,78]. Furthermore, the synthesized nanoparticles were cuboidal and orthorhombic in shape, ranging from 15 nm to 80 nm, respectively. The antibacterial activity was tested against five bacterial pathogens, i.e., *Escherichia coli*, *Salmonella typhi*, *Vibrio cholera*, *Pseudomonas aeruginosa*, and *Proteus mirabilis*. The phytosynthesized AgNPs showed good bactericidal activity at all concentrations (100 to 400 µg) against all five tested microorganisms. The nanoparticles exhibited a maximum zone of inhibition of 18 mm at 400 µL concentration against *V. cholera* and a minimum level of bacterial activity of 8 mm at 100 µg against *P. mirabilis*. Further studies need to be performed to examine the efficacy of the nanoparticles by determining the MIC values to quantify the results obtained by the group [127].

Another study conducted by Acharyulu and coworkers (2014) [107] demonstrated the synthesis of CuONPs using *Phyllanthus amarus* leaf extract. The CuONPs showed a SPR band at 285 nm, which is ascribed to Cu NPs crystalline structures according to the (JCPDS 45-0397) database. Moreover, the NP size was calculated to be between 22 nm and 50 nm using the Sherrer formula and SEM images, respectively. The group postulated that the bactericidal properties of CuONPs are characterized by the size, stability, and concentration of the NPs added to the inoculum solution. The antibacterial properties of the NPs showed a significant inhibition effect towards both the Gram-negative (*P. aeruginosa* and *E. coli*) and Gram-positive (*S. aureus* and *B. subtilis*) bacterial strains investigated in the study. In comparing the zone of inhibition study against the positive control (the antibiotic Rifampicin), the group observed that the CuONPs demonstrated an efficacy of about 55% more than the positive control used in this study. Moreover, the MIC showed that the NP’s efficacy was 22% higher than that of Rifampicin against Gram-negative bacteria and 32% more effective against the antibiotic under study. These findings emphasize the significance of phytonanotechnology’s effectiveness against pathogenic microorganisms, which needs more exploration [107].

Recently, Dharshini and coworkers (2021) [128] demonstrated the biosynthesis of FeNPs using *Phyllanthus reticulatus* leaf extract. The phytosynthesized FeNPs showed SPR bands at 229 nm, ascribed to FeNPs. Moreover, the synthesized NPs showed some functional groups assigned to phytochemical constituents responsible for reducing Fe ions to FeNPs. These findings are in agreement with other results mentioned above [67,77,129,130]. The size of the FeNPs was found to be in the range of 65 to 230 nm with an irregular spherical shape. Dharshini’s group studied the antimicrobial activity of the FeNPs against pathogenic bacteria (Gram-negative: *Proteus vulgaris*, *Vibrio chlorae*, *Shigellaflexneri*, *Salmonella typhi*, *Klebsiella pneumonia*, and *Pseudomonas aeruginosa*; Gram-positive: *Staphylococcus aureus*, *Streptococcus epidermis*). The activity of the NPs was measured using the zone of inhibition for different microorganisms. The NPs exhibited a maximum of 32 mm and a minimum of 25 mm of zone inhibition against Gram-negative bacteria.

In comparison, a maximum of 17 mm and a minimum of 15 mm for Gram-positive bacteria were observed. The group further investigated the antifungal properties of the FeNPs against (*Trichoderma viridae*, *Aspergillus niger*, *Aspergillus fumigatus*, and *Aspergillus flavus)*. The NPs demonstrated a maximum of 30 mm and a minimum of 18 mm zone of inhibition and about a 55% improvement compared to the crude extract alone. This significant improvement suggests that NPs enhance the antimicrobial properties of the extracts [128].

## 13. Phytonanoparticles for Anticancer and Antiviral Activity

Nowadays, nanotechnology has advanced applications that have expanded to cover a vast array of domains, especially in medicine. Metallic nanoparticles produced through phytochemical reduction have recently gained attention due to their pronounced applications. This rising interest in research on phytonanotechnology is due to its promising advantages over its physicochemical counterparts. In a study conducted by Maheswari and coworkers (2021) [121], they demonstrated the synthesis of TiO_2_ nanoparticles modified with *Plectranthus amboinicus*, *Phyllanthus niruri*, and *Euphorbia hirta*. The analysis showed that the NP size was between 4 and 15 nm in diameter, with a spherical shape.

Moreover, the optical density was observed between 264 and 335 nm depending on the phyto-modified TiO_2_ NPs. The group further tested the NPs for their antimicrobial activity against *Pseudomonas aeruginosa*, *Streptococcus mutans*, *Klebsiella pneumonia*, *Escherichia coli*, and *Staphylococcus aureus*. The anticancer activity and toxicity were determined against KB oral cancer cells and normal L929 cells. The NPs exhibited better antibacterial activity against *S. mutans*. In addition, the anticancer study demonstrated that all phyto-modified TiO_2_NPs showed good activity against the KB oral cancer cell lines. The group noticed that the *P. amboinicus-P. niruri* modified TiO_2_NPs exhibited great anticancer activity at various concentrations. The activity of these NPs, as shown with flow cytometry analysis, was due to the generation of the p53 protein, which exhibited the anticancer nature of these NPs.

The NPs showed no significant toxicity in normal cells. The minimum cell viability was recorded at 50 µg/mL. Furthermore, the group successfully demonstrated the excellent antimicrobial and anticancer activity of the *Plectranthus amboinicus-Phyllanthus niruri* modified TiO_2_NPs, which can be further used for therapeutic applications in the future [121].

A study conducted by Wang and coworkers (2021) [119] demonstrated the synthesis of gold nanoparticles using *Phyllanthus emblica* fruit extract. The NPs showed a SPR at 545 nm, ascribed to AuNP formation. An analysis with FTIR showed that the presence of phenols and flavonoids on the surface of the NPs suggests they may be responsible for the reduction of Au ions to AuNPs. The size and shape of the NPs were measured using DLS and TEM, which did not corroborate with each other. The DLS analysis reported an average 133 nm size, which was not uniform; however, with TEM, the average diameter was far too small compared to the one from the DLS analysis. This variation results from the available biomolecules around the AuNPs, which make them appear big. Wang’s group further investigated the cytotoxic activity of the synthesized NPs against normal and cancer cells. The groups observed that after hours of incubation of the cancer cells with the NPs, there was excellent visibility of the NP localization in the cancer cells, suggesting that the cells absorbed the NPs.

In addition, the NPs induced a significant decrease in cell viability in a dose-dependent manner. The half-maximal inhibition (IC50) of the NPs was measured at 80 µg/mL and 100 µg/mL against the AGS cancer cell lines. The group further investigated colony formation via microscopy; they observed that colony formation was significantly reduced in cells treated with 100 µg/mL of AuNPs compared to the positive control (cisplatin) used. This suggests that the anticancer activity of AuNPs inhibits colony formation without having toxic effects on normal cells. Lastly, the cell death mechanism was observed to be apoptotic cell death due to shrinkage of the cell morphology [119].

A study by Unni and coworkers (2014) [120] reported the synthesis of *Phyllanthus niruri* nanoparticles (*P. nir*-NPs). The nanoparticles were synthesized from whole plant methanol extract using the precipitation method, with the reaction solution containing 20 percent extract to reduce the hydrophobicity of the extract. The *P. nir*-NP mean size was 150 to 250 nm, and they were spherical. *Phyllanthus niruri* is well known for its potential effects on prostate cancer; therefore, Unni’s group investigated the efficacy of the *P. nir*-NPs against PC3 prostate cancer cell lines by the direct method. The study was carried out using a dose-dependent method, and the concentration ranged from 0.25 to 5.0 mg/mL. The group observed no noticeable activity at concentrations lower than 2 mg/mL; however, there was significant anticancer activity at concentrations of 2 mg/mL and more against the PC3 cancer cells. They attributed this to the presence of bioactive compounds like garlic acid, quercetin, and caffeoylquinic acid, which have been previously shown to have anticarcinogenic activities. However, the study did not specify whether the IC50 was determined to optimize and quantify their findings. Consequently, it is reported in the study that the nanoparticles induced significant cytotoxicity in the cancer cell line of choice. Furthermore, from the obtained results, the *P. nir*-NPs induced apoptotic cancer cell death [120].

Nguyen and coworkers (2020) [73] studied the synthesis of silver nanoparticles from *Phyllanthus urinaria* (*P. uri*-AgNPs), *Pouzolzia zeylanica* (*P. zey*-AgNPs), and *Scoparia dulcis* (*S. dul*-AgNPs). For this review, the focus is on *P. uri*-AgNP’s efficacy against fungal activity. The group observed that the size of the nanoparticles for *P. uri*-AgNPs was 28 nm. Moreover, the NPs SPR was observed between 400 and 550 nm, demonstrating the presence of AgNPs. They also reported that the mechanism of reduction for the formation of NPs in the plant extracts was an oxidation-reduction process, which agrees with the earlier reports tabled in this work.

Furthermore, the *P. uri*-AgNPs were tested for their antifungal activity against *A. niger*, *A. flavus*, and *F. oxysporum*. In this study, they observed that the proliferation of fungal cells was suppressed by the presence of AgNPs in a dose-dependent manner. At the same time, the extracts on their own did not influence cell proliferation under the same conditions. At lower concentrations (30 ppm), the *P. uri*-AgNPs did not show any significant antifungal activity against all tested fungal strains; however, at 30 and 45 ppm, there was a considerable amount of cell death induced by AgNPs. It can be concluded that the green synthesis of MNPs holds promise as a key technology in combating infectious diseases and malignant tumors [73].

## 14. Other Innovative Applications of Phytonanotechnology

In recent years, nano-based products have seen a wide growth in industrial applications in our day-to-day lives. To date, there are various commercial eco-friendly nanoproducts with high efficiency on the market [131]. For example, silica NPs, silver NPs, and platinum NPs have found use in various cosmetics and personal care products. They are used as active ingredients in products such as toothpaste, mouthwash, sunscreens, hair care products, perfumes, and anti-aging products. Moreover, silica and modified silica NPs have been used as excellent pesticide controls in the agricultural industry [132].

Metallic silver is a high heat-conducting material; due to this property, nano-silver is used in diverse mechanical devices. It is mainly used in instruments such as PCR lids and UV-visible spectrophotometers. Some parts of these instruments are made from nano-silver coating material, which is highly stable at high temperatures and does not interfere with the samples [133]. In the food industry, food products are prone to contamination with microorganisms during the processing, manufacturing, and shipping of raw materials. Therefore, metallic NPs have been used in biosensors to detect pathogens and monitor different stages of contaminants at low cost [133].

In the past few decades, high growth in antimicrobial resistance to antibiotics has been witnessed, and it has resulted in high mortality rates in humans. The re-emergence of pathogenic antibiotic-resistant Gram-negative and Gram-positive bacteria became a major public health concern globally [127,134,135]. Therefore, green NPs have found great use as inhibitory activity enhancers and as antimicrobial efficient agents [133].

Furthermore, in the agriculture sector, the current major drawback of livestock stock additives and nutrient supplements is to increase the production of livestock while maintaining product quality, protecting the environment from hazardous substances, and providing food security [111]. Nanotechnology can deliver novel vehicles for nutrient supplementation while improving the functionality of feed molecules, respectively. Additionally, minerals in the form of NPs bypass the intestinal wall and cells more easily due to their size, thus enhancing their bioavailability [102]. For example, the addition of ZnNPs to livestock feed has been found to improve immunity, increase growth, and increase milk production and reproduction in cows [112]. A study by Yang and Sun [136] reported a significant drop in diarrhea incidences by supplying a graded dose of ZnONPs in the feed diet. Moreover, Fondevilain (2010) [137] reported that the addition of AgNPs in animal feed enhanced antimicrobial efficacy and selectively combated potential pathogens. Therefore, NPs are a future nutrient tool in animal feed to improve production performance, enhance bioavailability, and increase the immunity of livestock [138]. These are a few attributes that brought about NPs’ influence in various sectors to improve our livelihood.

## 15. Future Innovative Prospects of Phytonanotechnology 

Recently, a wide range of limitations in conventional therapies, such as toxicity and high cost, have made it mandatory to develop and design novel drugs. Accordingly, the opportunity to apply various eco-friendly nanosized NPs in a greener way has opened doors for new frontiers in phytonanotechnology [120,122]. Over the past decades, advancements in nanotechnology have promoted industrial and biomedical applications of phytonanoparticles in industries including bioimaging, drug delivery and detection, etc. [121]. From various relevant studies, it is evident that bio-derived nanomaterials have been investigated in vitro, while there are limited data on their in vivo applications. Therefore, there is a great need for in vivo trial studies to be investigated to understand the in vivo toxicity mechanisms of phytonanoparticles through tests to generate data depicting nanomaterial behavior before clinical research [139,140]. Shortly, the application of plant-derived nanomaterials will see significant growth, which is expected to shed light on green nanotechnology’s long-term effects on animals, plants, humans, and the environment [141]. Some of the laboratory experiments by various research groups have shown beneficial effects of green nanomaterial applications in various sectors. However, there are limited data related to the use of the *Phyllanthus* genus as a capping and reducing agent for the biofabrication of phytonanoparticles. There is a lack of data in the literature regarding the use of the genus *Phyllanthae* as a potential plant material for the synthesis of cost-effective, nontoxic, bioavailable, and safe nanomaterials for various applications. Therefore, this review intends to shed light, bring a broad perspective, and highlight the need for further investigations in the future for the synthesis and production of various nanomaterials using the *Phyllanthus* genus.

## 16. Conclusions

This review presents information about the eco-friendly biosynthesis of metallic nanoparticles with particular stress on the understanding of underlying mechanisms for biosynthesis on NPs. It particularly emphasizes the biomolecules of plant extracts from the *Phyllanthus* genus that are involved in the reduction of metal ions to their NP forms and their consequent applications in various industrial sectors. Moreover, this review also highlights the significance of phytonanotechnology as a supplementary method for developing nanoparticles with bioactive properties over traditional methods. Moreover, we show the significance of the green synthesis approach as a feasible alternative method for phytonanoparticle production with enhanced activity against microorganisms and cancers alike. In addition, this review also demonstrates the potential of the genus *Phyllanthus* in reducing metal ions to their nanoparticle forms. The *Phyllanthus* family is known for its population of medicinal plants, and there are also significant findings by various groups that nanoparticles reduced with *Phyllanthus* lead to more great medicinal applications. Moreover, the synthesized NPs reported in this review demonstrated strong cell interaction with phytonanoparticles, leading to apoptotic cell death. However, more work needs to be performed based on phytonanotechnology using members of the *Phyllanthus* genus to unpack their potency as bactericidal, fungicidal, and anticancer agents.

## Figures and Tables

**Table 1:** Biosynthesis of NPs using *Phyllanthus* plant extracts and their antibacterial applications.

		Synthesis Conditions							Characterization	
Plant	NPs	Part of the Plant	Contact Time	Temp. (°C)	pH	Size (nm)	Morphology	Applications	Effective Conc. (MIC)	Ref.
*P. niruri*	Ag	Leaf	6 and 24 h	100	6 and 9	90 to 94	Spherical, triangular, cuboidal	N.S	N.S	[103]
*P. maderaspatensis* L.	Ag	Leaf	24 h	28	N.S	59 to 76	Spherical	N.S	N.S	[101]
*P. Pinnatus*	Ag	Stem	90	28	N.S	<100	Spherical	Antimicrobial activity on *B. subtilis*, *P. vulgaris*, *V. cholera*, *S. flexneri*, *M. smegmatis*, and *P. aeruginosa*	40 µL (N.S)	[102]
*P. emblica*	ZnO	Stem	2 h	60	N.S	15 to 25	Spherical	Antimicrobial activity on *S. typhi* and *K. phnemonea*	200 mg/mL (N.S)	[104]
*P. amarus*	Ag	N.S	15 to 25 min	60 to 70	7.4	51	Distorted spheres	Antibacterial and antifungal against *E. faecalis*, *K. pneumonia*, *E. coli*, *S. flexneri*, *S. typhi*, *P. mirabilis*, *P. aeruginosa*, *S. aureus*, *S. marcescens*, *A. niger*, and *C. albicans*	100 µg/mL (12.5 µg/mL)	[105]
*P. amarus*	MnO	N.S	1 h	R.T	6.8	40 to 50	Nanorods	N.S	N.S	[106]
*P. amarus*	CuO	Leaf	7 h	130	N.S	22 to 50	Spherical	Antibacteria activity against *S. aureus*, *B. subtilis*, *P aeruginosa* and *E. coli*	N.S	[107]
*P. amarus*	Ag	Leaf	3 d	R.T	N.S	N.S	Spherical and cuboidal	Antibacterial activity against *E. coli* and *B. subtilis*, and antioxidant properties	N.S	[108]
*P. acidas*	Ag	Leaf	24 h	25	N.S	200	Spherical	Antibacterial activity against *E. coli*	10 and 20 μg/mL (N.S)	[109]
*P. amblica*	Ag	Fruit	15 min	R.T	N.S	19 to 45	Spherical	Antimicrobial activity against *K. pneumoniae* and *S. aureus*	10 μg/mL (N.S)	[110]
*P. acidas*	Ag	Fruit	30 min to 9 h	100	N.S	10 to 46	Cuboidal	Anti-inflammatory effect against H_2_O_2_ exposed rat peritoneal macrophages	25 mg/mL (N.S)	[97]
*P. amblica*	ZnO	N.S	2 to 3 h	80	N.S	3 to 11	Quasi spherical	Antibacterial activity against *S. pyogenes*, *B. cereus*, *E. coli*, and *P aeruginosa*; antibiofilm activity	5 to 1000 μg/mL (50 μg/mL)	[111]
*P. amblica*	MgO	Fruit	4 h	R.T	N.S	27	Spherical	Antibacterial properties against *S. aureus* and *E. coli*	N.S	[112]
*P. amarus*	Ag	Whole plant	20	70	N.S	16 to 30	Spherical	Antimicrobial activity against 15 *P. aeruginosa* bacteria strains	13 to 100 μg/mL (6 to 13 μg/mL)	[105]
*P. acidas*	Au	Leaf and twig	N.S	R.T	N.S	81 to 94	Spherical and cuboidal	Bionano catalytic activity of α-amylase, cellulose, and xylanase.	Catalytic properties at 0.3 to 0.65 U/μg	[113]
*P. emblica*	Pd	Seed	3 h	60	6.8	30	Spherical	Antimicrobial activity against *B. subtilis*, *S. aureus*, and *P. mirabilis*. Cytotoxicity effects on HeLa cell lines and hemolytic activity	75 and 100 mg/mL (29, 27 μg/mL)	[114]
*P. emblica*	MgO	Fruit	10 min	120	N.S	100 to 200	Porous and spongy	Antimicrobial activity against *P. aeruginosa*, *S. aureus*, *A. baumannii*, *E. coli,* and *K. pneumoniae*. Degradation of Evans blue	12.5 to 100 mg/mL (50 mg/mL)	[115]
*P. emblica*	Ag	Fruit	30 min	R.T	2 to 10	10 to 70	Spherical	Antimicrobial activity against *S. aureus*, *B. subtilis*, *E. coli* and *K pneumonia*	N.S	[70]

NPs: nanoparticles, Temp.: temperature, N.S: not specified, R.T: room temperature.

**Table 2:** Biosynthesis of NPs using *Phyllanthus* plant extracts and their anticancer and antifungal applications.

		Synthesis Conditions				Characterization				
Plant	NPs	Part of the Plant	Contact Time	Temp. (°C)	pH	Size (nm)	Morphology	Applications	Effective Conc. (IC_50_)	Ref.
*P. fraternus*	Ag	Leaf	72	25	N.S	40 to 50	Spherical and distorted shape	In vitro anticancer activity against HepG-2 and MCF-7 breast cancer cell lines	0.97–250 µg/mL (62.5, 125 µg/mL)	[116]
*P. emblica*	Fe	Fruit	12 h	60	N.S	4 to 15	Spherical	Anticancer activity against human lung cancer cell line A549	50–1000 µg/mL (105, 360 µg/mL)	[117]
*P. maderaspatensis* L.	Ag	Root	24 h	N.S	N.S	3 to 14	Spherical	Antioxidant, antibacterial activities against *A. aureus*, *B. subtilis*, and *E. coli*, and anticancer activities against MCF-7 breast cancer cell line	50–250 µg/mL (67 µg/mL)	[118]
*P. emblica*	Pd	Seed	3 h	60	N.S	30	Spherical	Hemolytic activity, antimicrobial activity against *P. aeruginosa*, *B. subtilis*, *S. aureus*, and *P. mirabilis*, and anticancer activity against HeLa cell lines	150 µg/mL (15–50 µg/mL)	[114]
*P. emblica*	Au	Fruit	N.S	R.T	N.S	5 to 60	Spherical, triangular, and polygonal	Anticancer activity against human gastric carcinoma cell line AGS	50–100 µg/mL (80 µg/mL)	[119]
*P. niruri*	P.n-NPs	Whole plant	24 h	R.T	N.S	150 to 250	Spherical	Anticancer activity against prostate cancer	2–5 mg/mL (N.S) (mg/mL)	[120]
*P. niruri*	TiO_2_	Leaf	12 h	100	N.S	8 to 11	Spherical	Anticancer activity of modified TiO_2_ nanoparticles against KB oral cancer cell lines	10–50 µg/mL (N.S)	[121]

NPs: nanoparticles, Temp.: temperature, N.S: not specified, R.T: room temperature.

## Data Availability

Data is openly available and no new data were created for this study.
